# Cytokine and Chemokine Concentrations as Biomarkers of Feline Mycobacteriosis

**DOI:** 10.1038/s41598-018-35571-5

**Published:** 2018-11-23

**Authors:** C. O’Halloran, L. McCulloch, L. Rentoul, J. Alexander, J. C. Hope, D. A. Gunn-Moore

**Affiliations:** 1 Royal (Dick) School of Veterinary Studies and The Roslin Institute, University of Edinburgh, Easter Bush Campus, Edinburgh, Scotland EH25 9RG UK; 2 UK Dementia Research Institute, Edinburgh Medical School, University of Edinburgh, Edinburgh, Scotland EH16 4SB UK; 3 MilliporeSigma (a Division of Merck KGaA, Darmstadt, Germany), 3050 Spruce Street, St. Louis, MO USA; 4 Waltham Centre for Pet Nutrition, Leicestershire, UK

## Abstract

Mycobacteriosis is an emerging zoonotic disease of domestic cats and timely, accurate diagnosis is currently challenging. To identify differential cytokine/chemokine concentrations in serum/plasma of cats, which could be diagnostic biomarkers of infection we analysed plasma/serum from 116 mycobacteria-infected cats, 16 healthy controls and six cats hospitalised for unrelated reasons was analysed using the Milliplex MAP Feline Cytokine Magnetic Bead multiplex assay. Three cytokines; sFAS, IL-13 and IL-4 were reduced while seven; GM-CSF, IL-2, PDGF-BB, IL-8, KC, RANTES and TNF-α were elevated in mycobacteria-infected cats compared to healthy controls. However, IL-8 and KC concentrations were not significantly different from cats hospitalised for other reasons. Elevations in TNF-α and PDGF-BB may have potential to identify *M*. *bovis* and *M*. *microti* infected cats specifically while GM-CSF, IL-2 and FLT3L were increased in MTBC infected cats. This study demonstrates potential use of feline tuberculosis as a spontaneously occurring model of this significant human disease. Cytokine profiling has clear diagnostic potential for mycobacteriosis of cats and could be used discriminate tuberculous from non-tuberculous disease to rapidly inform on zoonotic risk. Future work should focus on the in-field utility of these findings to establish diagnostic sensitivity and specificity of these markers.

## Introduction

Tuberculosis (TB) has been the cause of considerable morbidity and mortality in the human population for centuries, and remains the leading cause of death from any single infectious agent (*Mycobacterium (M*.*) tuberculosis*) worldwide^1–3^. Historically, the significance of this disease has resulted in extensive biomedical research interest and, due to the growing threat of drug resistance, TB is a major focus of collaborative global healthcare investment^4–8^. Meanwhile, TB in domestic cats receives relatively little research attention, despite being more common and clinically significant than previously thought^9–11^. In the UK, approximately 1% of all feline biopsies submitted for routine histopathological analysis show changes consistent with mycobacteriosis and a third of these contain Ziehl-Neelsen (ZN) positive organisms when stained, with morphology indicative of the presence of mycobacteria^10,12^.

The various mycobacterial species that have been identified in companion animals, including cats, can be grouped into the same two major categories as human mycobacterial disease; those belonging to the *Mycobacterium tuberculosis-*complex (MTBC) and the non-tuberculous mycobacteria (NTM, also referred to as ‘atypical mycobacteria’ or ‘mycobacteria other than tuberculosis’, MOTT)^11,13–19^.

The MTBC consists of ten highly genetically related species of mycobacteria which are capable of causing TB in both man and other animals, and are some of the oldest recorded zoonotic diseases known to both human and veterinary medicine^20–26^. All member species of the complex share identical sequences across the 16s rRNA gene and 99.5% sequence homology across the remainder of the genome^27^. The most discriminating features between the species at the nucleotide level are genomic deletions, termed regions of difference (RD)^28–31^. The MTBC organisms which infect cats, *Mycobacterium microti* and *Mycobacterium bovis*, differ by RD1^12,32–34^. This region is absent from *M*. *microti* but present in all other MTBC organisms^28^. It has been shown to encode a variety of molecules which act as virulence factors which are also immuno-dominant proteins such as; early secreted antigenic target-6KDa (ESAT-6) and culture filtrate protein-10KDa (CFP-10)^35–37^.

In companion animals, such as cats, MTBC infections pose a potential zoonotic risk to their owners; additionally these cases may act as a potential source of environmental contamination with mycobacteria such as *M*. *bovis*, a pathogen of major animal health significance in the UK and other countries as the causative agent of bovine tuberculosis (bTB)^38–41^.

In stark contrast to human TB cases, the majority of feline TB cases present with localised nodular cutaneous disease, frequently with a degree of ulceration and occasionally with a draining sinus tract^9,13,14,34^. The lesions are typically distributed around the face, extremities and tail base – the so-called “fight and bite sites”^15,34^. Skin lesions may be accompanied by a localised or even generalised lymphadenopathy, or lymphadenopathy (usually of the submandibular, pre-scapular or popliteal nodes) may be the only presenting sign, termed an incomplete primary complex^14,33,34^.

Pulmonary lesions do occur in cats, but rarely result from bacteria being inhaled and causing typical tubercle formation in the lungs and hilar lymph nodes^9,14^. Much more commonly, pulmonary disease is secondary to the putative haematogenous spread of bacteria from the site of inoculation in the skin^12,14^. This generates a diffuse interstitial pattern of disease which eventually becomes bronchial and is clinically observable as progressive dyspnoea followed eventually by a soft productive cough^14,34^. Radiographically this presents differently from primary pulmonary infection which more frequently causes the “classical” tuberculous cavitating lesions^34^. Disseminated disease can cause a range of clinical signs including hepato-splenomegaly, pleural and pericardial effusions, generalised lymphadenopathy, weight loss and pyrexia^9,13,16,33,34^.

With such variable clinical presentation and non-specific clinical signs, diagnosing feline mycobacterial infections rapidly and accurately is challenging. Mycobacterial culture conducted by the Animal and Plant Health Agency (APHA), is currently the recognised ‘gold standard test’ for the diagnosis of mycobacterial disease in UK companion animals; however, it has several limiting features. Firstly, it has been shown to have a relatively poor sensitivity of ~50%, secondly it requires weeks from the time that a contaminated tissue biopsy is submitted for results to be obtained *e*.*g*. the average culture time is 12–16 weeks for *M*. *microti* - the most frequently cultured organism^9,34,42^. During this time, if untreated, a patient will remain a potential source of infection to both its owner and the local environment.

As such, alternative diagnostic tests have been developed which aim to reduce the amount of time between clinical presentation and a definitive diagnosis being reached. Interferon-gamma (IFN-γ) release assays (IGRA) were developed on the principle of quantitatively evaluating IFN-γ production by peripherally circulating antigen-specific effector T-memory cells upon *in vitro* stimulation, in order to aid the diagnosis of bTB in cattle^43–46^. These have subsequently been adapted to identify both active and latent TB in human patients with both greater sensitivity and specificity than the previously utilised tuberculin skin test^47–49^. Where intra-dermal testing has been shown to be of unreliable clinical utility in the cat, the IGRA has been used successfully^14,45^.

IGRAs have several advantages over other diagnostic techniques; they are significantly quicker at generating results than culture and are cheaper than many commercially available PCR and subsequent sequencing methods, they are also relatively non-invasive requiring only a single peripheral blood sample^45,48^. Unlike intra-dermal skin testing, they can be repeated if necessary as conducting the assay does not alter the systemic immune response^44,50,51^. In 2008, an adapted methodology of the IGRA was validated for diagnostic use in cats; this assay has a reported 100% sensitivity to indicate MTBC infection^45^.

Recently, within the field of human diagnostics of mycobacterial diseases, and of TB in particular, there has been a marked increase in research focussed on the identification of circulating cytokine biomarkers for the diagnosis of both active and latent MTBC infections in humans^52–57^. The goal of such assays is to cheaply, sensitively and rapidly identify infected and infectious individuals, in order to combat what remains one of the most incident infectious diseases of man^58^. Such studies have utilised multiplex cytokine assays, most commonly Luminex xMAP technologies, with promising results^54,59,60^. Studies of multiple assays have found circulating cytokine concentrations and combinations of cytokines which can sensitively and specifically diagnose both active and latent TB in humans^52–57^. For example; CXCL10 and CCL2 plasma concentrations can be combined with circulating levels of IFN-γ to more accurately discriminate between active and latent TB states, whilst the quantification of vascular endothelial growth factor can help differentiate patients with TB pleural effusion from those with neoplastic pleural effusions^57,61^. Similar assays have also been used successfully in the study of a limited number of companion animal diseases, including canine lymphoma and feline cystitis^62–65^.

As the timely and accurate diagnosis of feline mycobacteriosis, and the identification of the causative species is currently challenging, the aim of this study was to evaluate whether cytokine profiling from peripheral blood samples of infected cats demonstrated the same clinical utility as has been shown for humans. We therefore hypothesised that a number of cytokines would be differentially detectable in cats infected with mycobacterial infections when compared to both healthy controls and sick cats hospitalised for other reasons. We further hypothesised that the cytokine response would differ according to the species of mycobacteria present in the patient.

## Materials and Methods

### *Mycobacteria* spp. infected cat blood sample collection

Blood samples analysed in this study consisted of either heparinised plasma or separated serum. Archived remnant samples obtained opportunistically by vets and donated by the cats’ owners were used in this project. Cats diagnosed with mycobacterial infection by histological identification of (pyo)granulomatous inflammation in lesion biopsy material with the presence of acid-fast bacilli morphologically indicative of mycobacteria were considered eligible for inclusion in this study. Private veterinary surgeons who contacted the Royal (Dick) School of Veterinary Studies for clinical assistance with case management were asked to retain any blood samples remaining after diagnostic procedures had been performed. With the owner’s consent, these samples were then sent directly to the University of Edinburgh where they were retained at −80 °C prior to analysis of chemokine and cytokine concentrations.

Cats were excluded from the study if, prior to blood sample collection, they had been known to be treated with immunomodulatory medications e.g. non-steroidal anti-inflammatories (NSAIDs), chemotherapeutic agents or corticosteroids within 14 days of sample collection. Cats were additionally excluded if they were pre-treated with antibiotics with efficacy against mycobacteria, including fluoroquinolones, macrolides/azides or doxycycline within the same time period. Cats were not excluded if they had been treated with antimicrobial agents if these would be ineffective against mycobacteria, such as a penicillin or cephalosporin. Similarly, cats were not excluded if they had been treated with non-immunomodulatory analgesic medications e.g. opioids.

This study was conducted following approval from the School of Veterinary Medicine Ethical Review Committee at the University of Edinburgh; all relevant guidelines and regulations were adhered to throughout.

### Speciation of mycobacteria infecting case animals

All cats included in this study had positive histological diagnoses of mycobacterial infection as outlined above. Of these, cases which had undergone additional IGRA, PCR or culture testing were sub-classified by the species of infecting organism identified.

Cases were defined as being infected with an MTBC mycobacteria if the cat had an IGRA test which showed an antigen specific IFN-γ response biased to purified protein derivative from *M*. *bovis* (PPDB) above that generated in response to purified protein derivative from *M*. *avium* (PPDA). The MTBC cases which additionally responded to the ESAT-6/CFP-10 peptide combination were classified as *M*. *bovis* infected. Cats which did not respond to the ESAT-6/CFP-10 peptide cocktail but showed a PPDB biased response were defined as being infected with *M*. *microti*. Cats with a positive IGRA test that did not meet the criteria for MTBC infection were classified as being infected with non-tuberculous mycobacteria. Both the test method and interpretation have previously been described by Rhodes *et al*.^45^.

In addition to IGRA testing, the results of mycobacterial culture from tissue biopsies were used, where available, when conducted by APHA and/or PCR results were used to speciate infections when conducted by Leeds University Teaching Hospital Mycobacterial Reference Laboratory.

### Collection of control cat blood samples

Remnant serum samples from healthy control cats were kindly gifted from the Waltham Centre for Pet Nutirtion. At the time of sampling each control animal underwent an annual routine health check comprising compete physical examination by the attending veterinary surgeon, plus full haematological testing and serum biochemical analyses. All cats were required to have findings for these tests within normal limits to be included as a control animal in this study. Separated serum were aliquoted, and sent directly to the University of Edinburgh and stored −80 °C prior to analysis of chemokine and cytokine concentrations.

### Collection of hospitalised cat blood samples

Remnant serum was obtained from cats Hospital for Small Animals, at the Royal (Dick) School of Veterinary Studies, University of Edinburgh. These cats had no clinical signs consistent with mycobacterial disease and were only included if an alternative definitive diagnosis had been reached. Once obtained, samples were kept at 4 °C and assayed within 12 hours.

Cats were excluded from this group for any one or more of the following: a diagnosis of stage V neoplasia (*i*.*e*. bone marrow involvement was confirmed), myelodysplastic disorder, exogenous retroviral (FIV/FeLV) infection or if they had received treatment with any immunomodulatory medicines including NSAIDs, corticosteroids or chemotherapeutic agents.

### Cytokine and chemokine measurements

Cytokine and chemokine concentrations were measured in all of the samples using a commercial, feline specific, antibody-coated microsphere-based multiplex cytokine immunoassay shown to be able to quantify 19 cytokines contemporaneously using 25 ul of each patient’s serum (FCTYOMAG-20K MILLIPLEX MAP Feline Cytokine/Chemokine Magnetic Bead Panel, Premix 19 Plex kit, MERCK Millipore Corporation, Billerica, MA, USA).

The following cytokines were measured: sFas, Flt-3 ligand, GM-CSF, IFN-γ, IL-1β, IL-2, IL-4, IL-6, IL-8, IL-12-(p40), IL-13, IL-18, KC, MCP-1, PDGF-BB, RANTES, SCF, SDF-1, TNF-α. All samples, standards and quality controls were assayed in accordance with the manufacturer’s instructions. All samples were assayed in duplicate and mean values analysed. Overnight incubation at 4 °C and a magnetic plate washer were utilised. The plates were read with a multiplex plate reader and companion software. All cytokine and chemokine concentrations are reported in pg/mL.

### Statistical analysis

Statistical analysis was performed using commercially available statistical software (GraphPad Prism 7.0). Mean values of duplicate samples for each cat were used for analysis. For the purposes of statistical analysis, values that fell below the limit of detection of the assay were assigned a concentration of 0 pg/mL.

Initially, to compare all mycobacteria positive samples (n = 116) to healthy control cats (n = 16), the D’Agostino & Pearson omnibus test was used to confirm a Gaussian distribution of the control group data for each of the cytokines/chemokines measured. As no reference intervals currently exist for these biomarkers in the cat, these were generated from the healthy control cat population to include values 1.96 standard deviations above and below the mean of the control group (*i*.*e*. to encompass ~95% of the healthy population) for each cytokine/chemokine measured. The mean concentration, standard deviation and reference interval (RI) generated for each molecule measured are shown in Supplementary Table I.

For mycobacteria-infected cats, 95% confidence intervals around the mean were generated by logarithmic-transformation of the cytokine values so that the data conformed to a Gaussian distribution. On this basis, a 95% confidence interval was then generated for the transformed data which was then reverse transformed to give the confidence intervals provided in Supplementary Table 1.

Cytokine levels were considered to be statistically significantly different between mycobacteria-infected and healthy control cats if there was no overlap between the RI generated from the control data and confidence intervals of data generated from infected cats (shown in bold in Supplementary Table I).

A second comparison was made between mycobacteria-infected, healthy control cats and hospitalised cats. Confidence intervals for the cytokine levels of hospitalised cats were generated in the same way as described for mycobacteria-infected cats and are shown in Supplementary Table 1. Again, cytokine levels were considered to be significantly different between groups if there was no overlap between the RI generated from the control data and confidence intervals of data generated from sick cats or mycobacteria infected cats.

A sub-group analysis was conducted to compare healthy control (n = 16), *M*. *bovis* infected (n = 22), *M*. *microti* infected (n = 43) and NTM infected cats (n = 15) with each other. Of these four groups, only cytokine concentrations within the healthy control group followed a Gaussian distribution as assessed by D’Agostino & Pearson omnibus test. Therefore, a Kruskal-Wallis test by ranks was used to determine if differences existed between the groups for each cytokine measured. Where significant differences were found (defined as p < 0.05), each group was compared in a consecutive pairwise manner using Mann-Whitney U tests with *post-hoc* Bonferroni correction applied to accommodate for multiple comparisons. Resultantly, a value of p ≤ 0.003 was considered statistically significant for sub-group analyses. The median, range and 95% confidence interval for the cytokine concentrations measured within each sub-group of mycobacteria-infected cats are shown in Supplementary Table II.

## Results

### Patient characteristics

All cats included in this study were adult cats from the UK. In total, serum/plasma from 116 cases of feline mycobacterial disease met the inclusion criteria for the study, and serum from 16 healthy control cats and six hospitalised cats was also analysed.

Within the group of cats with clinical mycobacteriosis, the infecting organism was speciated based on IGRA, culture and/or PCR for 80 of the 116 (69.0%) cats. Of these, 22 (27.5%) were infected with *M*. *bovis*, 43 (53.8%) were infected with *M*. *microti* and the remaining 15 (18.7%) infected with NTM.

For the cats hospitalised for independent reasons the final diagnoses were established as hyperthyroidism (n = 2), congestive heart failure due to hypertrophic cardiomyopathy, hepatic lipidosis, lysosomal storage disease, chronic kidney disease and diabetes mellitus.

### Cytokine and chemokine concentrations

#### All mycobacteria-infected cats compared to healthy control cats

**Figure 1:**
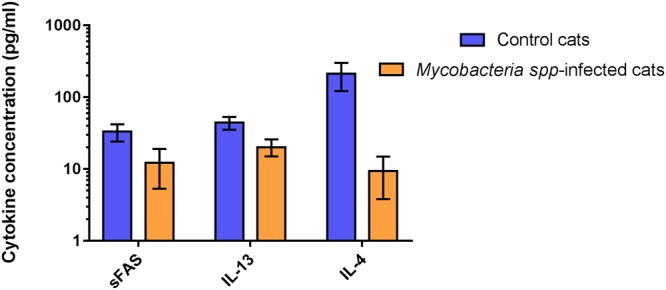
Cytokines found to have significantly reduced concentrations in the peripheral blood of cats with mycobacterial disease compared to healthy controls. Data are shown as the median for the group with error bars indicting the 95% confidence interval.

**Figure 2:**
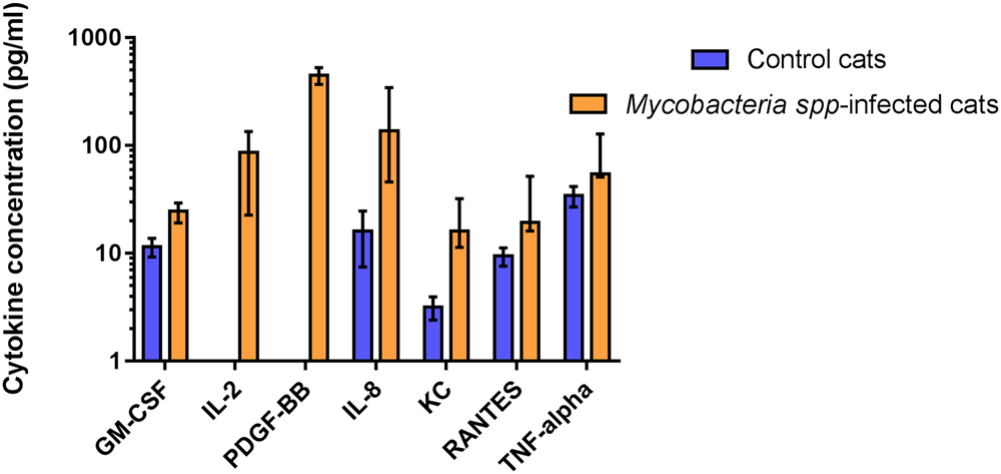
Cytokines found to have significantly increased concentrations in the peripheral blood of cats with mycobacterial disease compared to healthy controls. Data are shown as the median for the group with error bars indicting the 95% confidence interval.

The mean/median and standard deviation/range for each cytokine concentration for each of these groups is shown in Supplementary Table I. Three cytokines; sFAS, IL-13 and IL-4 were found to be significantly reduced in the mycobacteria-infected group compared to healthy control cats (Fig. 1). By contrast, seven cytokines; GM-CSF, IL-2, PDGF-BB, IL-8, KC, RANTES and TNF-α were present in significantly elevated concentrations within the peripheral circulation of mycobacteria infected cats when compared to healthy control animals (Fig. 2).

There was no significant difference between the two groups in their circulating concentrations of Flt-3 ligand, IFN-γ, IL-1β, IL-12 (p40), IL-6, IL-18, SDF-1, MCP-1, or SCF.

#### Comparison of all mycobacteria-infected cats to hospitalised cats and controls

To begin to assess the specificity of changes in cytokine concentrations between feline mycobacteriosis patients and healthy controls, serum from six hospitalised cats was analysed and compared to both of these groups, data shown in Supplementary Table 1.

**Figure 3:**
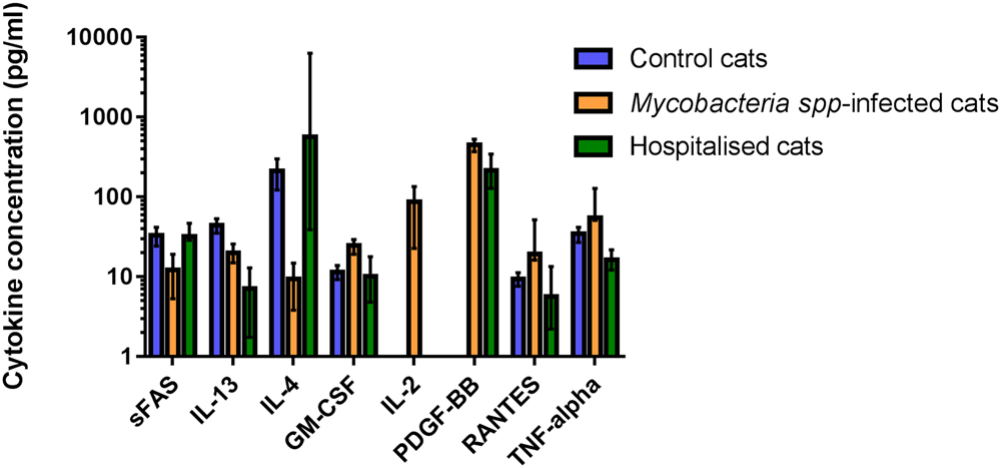
Cytokines found to have significantly altered concentrations in the peripheral blood of cats with mycobacterial disease compared to healthy controls and/or cats hospitalised for other reasons. Data are shown as the median for the group with error bars indicting the 95% confidence interval.

The cytokines GM-CSF, IL-2, PDGF-BB, RANTES and TNF-α were found at significantly elevated concentrations, whilst IL-4 and sFAS were detected at reduced concentrations in mycobacteria infected cats compared to both hospitalised non mycobacteria-infected cats and healthy controls (Fig. 3).

**Figure 4:**
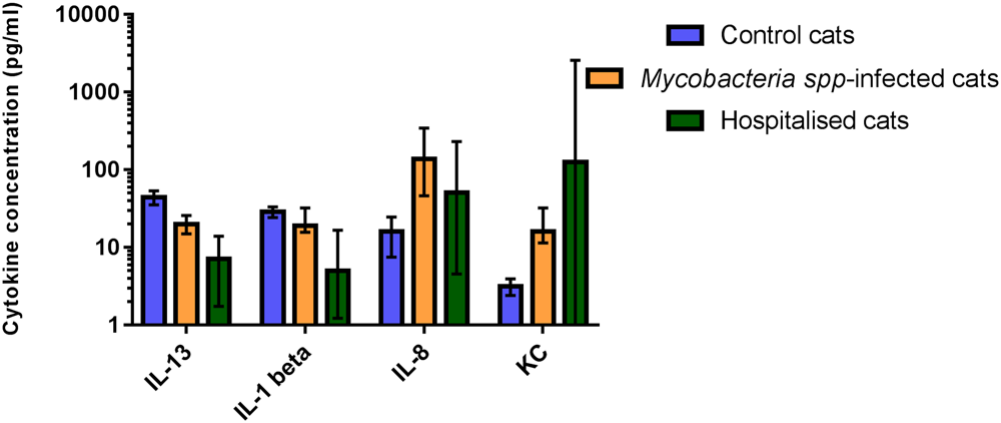
Cytokines found to have significantly altered concentrations in the peripheral blood of cats with mycobacterial disease compared to healthy controls and/or cats hospitalised for other reasons. Statistically significant differences between groups were determined by Kruskal-Wallis test by ranks (P < 0.05), each group was compared in a consecutive pairwise manner using Mann-Whitney U tests with *post-hoc* Bonferroni correction applied to accommodate for multiple comparisons (P ≤ 0.003). Data are shown as the median for the group with error bars indicting the 95% confidence interval.

The concentrations of IL-13 and IL-1β were found to be significantly reduced in hospitalised cats when compared to both control cats and those with mycobacterial infections (Fig. 4). Whilst differences in the concentrations of IL-8 and KC were observed between healthy cats and those infected with mycobacteria, there was no difference in the concentrations of these cytokines between cats infected with mycobacteria and cats hospitalised for unrelated conditions (Fig. 4).

#### Comparison of cats infected with different mycobacterial species

**Figure 5:**
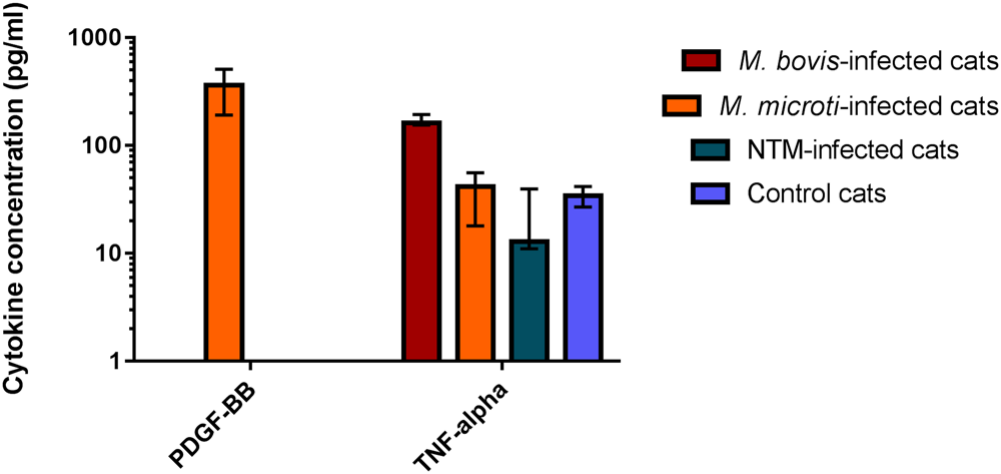
Cytokines found to have significantly altered concentrations in the peripheral blood of cats with mycobacterial disease due to *M*. *bovis* or *M*. *microti*, respectively, compared to healthy controls and cats infected with non-tuberculous mycobacterial (NTM) species. Data are shown as the median for the group with error bars indicting the 95% confidence interval.

**Figure 6:**
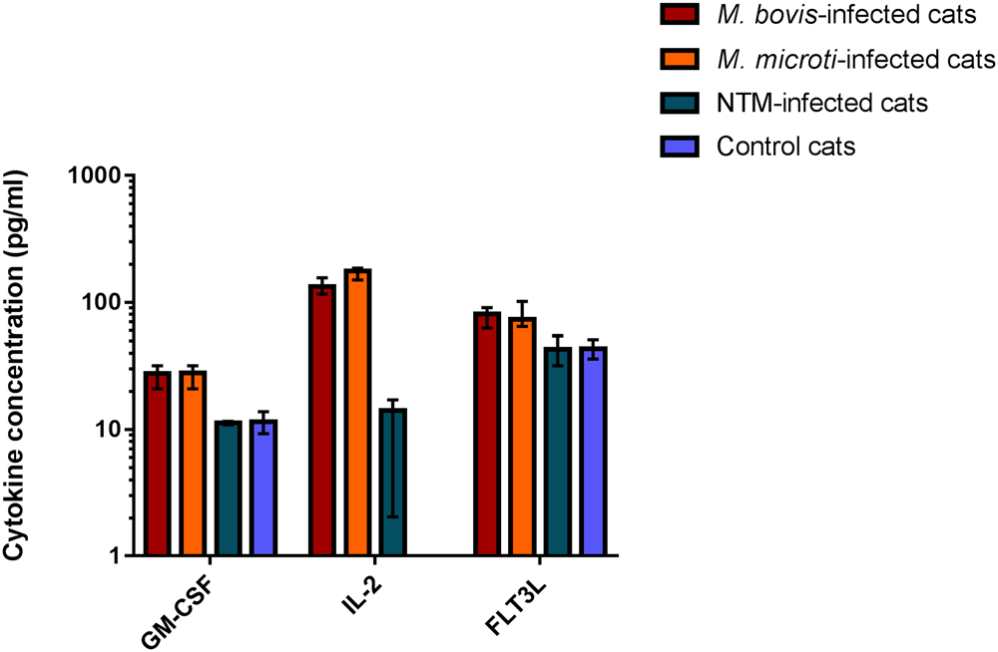
Cytokines found to have significantly altered concentrations in the peripheral blood of cats with mycobacterial disease due to TB-complex infections (*M*. *bovis* or *M*. *microti* respectively), compared to healthy controls and cats infected with non-tuberculous mycobacterial (NTM) species. Data are shown as the median for the group with error bars indicting the 95% confidence interval.

Of the 116 cats enrolled in the study, 80 had their mycobacterial infections definitively speciated to one of three causative agents, or group of causative agents; *M*. *bovis*, *M*. *microti* and NTM infections. These groups were compared to one another and also against the healthy control group. This analysis revealed the concentration of TNF-α to be significantly increased in the *M*. *bovis*-infected group when compared to all other groups; while the concentration of PDGF-BB was found to be significantly increased in cats infected with *M*. *microti* compared to all remaining groups (Fig. 5). Concentrations of GM-CSF, IL-2 and Flt3-L were significantly increased in both MTBC groups (*M*. *bovis* and *M*. *microti*) when compared to the remaining two groups (Fig. 6).

The group of cats infected with NTM species had a significant reduction in the concentration of MCP-1 in comparison to the healthy controls (data not shown).

Concentrations of cytokine IL-8 were increased in all three mycobacteria-infected groups when compared to healthy control animals (data not shown), with the greatest increase observed in the cats with NTM infections, though this trend was not statistically significant (p = 0.07).

## Discussion

Current routine clinical methods for the diagnosis of feline mycobacterial disease, and tuberculosis in particular, have a number of drawbacks. Multiplex immunoassays have proven to be sensitive, specific, rapid, require very small sample volumes, and show a broad analytical and dynamic range^66–70^. The benefits of the multiplex assay approach used in this study include more rapid availability of results than currently achievable with culture or IGRA, and that this analysis requires only a small volume of serum or plasma to analyse (25 ul)^66–70^.

This population of cats consisted of adult cats resident in the UK; previous studies have shown that male cats are over-represented in the population of cats presenting with mycobacterial infections and cats are usually younger when they present with *M*. *bovis* compared to *M*. *microti* infections^13,34^. It was not possible for us to determine if any such bias existed within our study population as signalment data was infrequently reported to us by primary veterinary clinicians. The reasons for this are unclear but it may be a reflection of the predominately outdoor and semi-feral lifestyles of many of these cats meaning that there is limited information definitively known about them.

All samples used in this study were opportunistically obtained remnants collected as part of other diagnostic investigations and processed by primary veterinary clinicians. This approach meant that samples could be sourced ethically and provides demonstrable ‘proof of concept’ that such methodology is potentially practicable and achievable within a clinical setting. Due to the remnant nature of samples analysed in this study, one must be cognisant of the possibility that post-collection processing may not have been consistent across samples e.g. storage temperature, which may have influence on individual findings^71,72^. However, it seems reasonable to presume that such adverse effects would not impact any one group of cats in this study in particular, but occur randomly throughout the dataset generated and so the results obtained in this study are still (clinically) interpretable. Similarly, due to the opportunistic nature of sample collection, this study included two different types of sample media; serum and heparinised plasma. Previous work with the same commercially available multiplex assay kit has demonstrated strong correlation between cytokine/chemokine concentrations obtained in paired serum and heparinised plasma samples taken from the same cat^70,73^, and so again it would seem the results obtained in this study remain reliable and interpretable.

The inflammatory cascade induced by mycobacterial infection is complex and is poorly characterised in the cat. The hallmark lesion of mycobacterial infections, including TB, is the granuloma which is a compact, organised aggregate of immune cells formed as part of the host immune response to the persistent stimuli of the pathogen^15,74–76^. Upon phagocytosis, inhibition of phagosome-lysosome fusion enables the intracellular survival of mycobacteria within these phagocytes^77^. The internalised bacilli are then believed to stimulate the infected macrophage to invade the local tissues^78,79^. The stimulation of macrophage Toll-like receptors (TLRs) by mycobacteria induces the production of numerous cytokines, predominantly TNF-α, which drives the recruitment of mononuclear cells and neutrophils from surrounding blood vessels. In particular, there is recruitment of activated T-cells which interact with activated macrophages and secrete IFN-γ leading to mycobacterial killing^80–84^. Additionally, each group of recruited cells also release their own assortment of cytokines and chemokines which perpetuate the inflammatory cascade and the formation of a stable granuloma structure^81,85^.

In this study, we demonstrated that changes in the concentrations of eight critically important cytokines gave a sensitive and specific indication of mycobacterial infection in this study population. Elevated concentrations of the cytokines GM-CSF, IL-2, PDGF-BB, IL-8, KC, RANTES and TNF-α in mycobacteria infected cats with concurrent reductions in sFAS, IL-13 and IL-4 are suggestive of a pro-inflammatory process occurring in these patients, which is dominated by the recruitment and maturation of monocyte-macrophage lineage cells, the recruitment of cytotoxic T-cells, the proliferation of fibroblasts and the suppression of humoral immunity.

Granulocyte-macrophage colony-stimulating-factor (GM-CSF) is a critically important cytokine involved in the innate immune response against mycobacteria, specifically via influencing macrophage and dendritic cell function^86–92^. GM-CSF deficient mice are extremely susceptible to mycobacterial disease and their macrophages fail to effectively inhibit intracellular pathogen replication^90^. Similarly, humans with detectable titres of anti-GM-CSF auto-antibodies show an increased susceptibility to pulmonary tuberculosis compared to healthy controls, whilst GM-CSF immunotherapy can contribute to the resolution of disease due to drug-resistant *M*. *tuberculosis*^93,94^. GM-CSF activated cells, principally macrophages, go on to propagate the inflammatory cascade in particular via the production of TNF-α^86^.

GM-CSF has been shown by previous multiplex cytokine analyses to be critical in the early response and possible clearance of *M*. *tuberculosis* in humans and mice, so that the use of recombinant GM-CSF as an adjunctive therapy for TB has been posited; the findings of this study suggest that this may also be of benefit to feline patients.

TNF-α is an agonist of both nuclear factor (NF)-kB and mitogen-activated protein kinase (MAPK) which lead to downstream upregulation of pro-inflammatory immune functions including but not limited to; increased cell adhesion (e.g. giant cell formation) and macrophage apoptosis which are beneficial to the control of mycobacterial infections^95–97^. One such downstream effect is the increased expression and secretion of chemokines including RANTES and interleukin IL-8^98–100^. These molecules are major drivers in the recruitment and migration of leukocytes towards an inflammatory focus^98–100^. Additionally, RANTES can combine synergistically with other locally secreted cytokines, such as IL-2 released from T-cells and IL-12(p40) released from pathogen-activated antigen-presenting cells e.g. mature dendritic cells, in order to stimulate the activation and proliferation of natural killer (NK) cells, which ultimately leads to a local increase in mycobactericidal activity^98–100^.

TNF-α, one of the most studied cytokines in the human mycobacterial response, has been repeatedly shown to be of significant diagnostic utility for mycobacterial infections in humans, with concentrations varying between TB and non-TB patients as well as being discriminatory between active and latent infections and generally being found to decline with successful therapy^53–56^. Similarly, a number of patients receiving TNF-α antagonists such as infliximab have been reported to contract and even die from mycobacterial infections^101–103^. In this study, it is notable that this cytokine was the only one to be uniquely indicative of infection with *M*. *bovis*. This is potentially due to the secretion of RD1 proteins during the course of these infections generating a more pro-inflammatory state in *M*. *bovis* infected cats. The possibility that cats infected with mycobacteria and with elevated TNF-α could have more severe or active disease and hence pose a more serious zoonotic threat to humans is one that warrants further investigation.

An essential step in the formation of an effective granuloma structure is fibroblast proliferation, mediated at least in part by the local production of PDGF-BB^104^. Similar to TNFα, it has recently been demonstrated that circulating levels of this cytokine are elevated in human patients with active pulmonary tuberculosis, and that these levels decline significantly over a six month course of therapy^54^. However, in contrast to TNFα, within our feline population, PDGF-BB was only detected at significant levels in cats infected with *M*. *microti*. This may indicate a difference in the immune response by cats to this species of mycobacteria, which was used as the human vaccine strain prior to the availability of *M*. *bovis-*BCG, and may therefore be considered attenuated^105^. Future analysis and comparison of the granuloma structures of *M*. *microti* and *M*. *bovis* infected cats may show physical manifestations of such differences, and may be useful to identify prognostic features of relevance to feline disease.

The Fas/Fas-ligand (FasL) system is an important molecule for immunological regulation; specifically it maintains T-cell homeostasis by the induction of apoptosis in order to limit T-cell expansion following antigenic stimulation^106,107^. It has been reported that mycobacteria exploit this system in order to subvert and evade the adaptive immune response of an infected animal by generating an immune-privileged niche^106,108^. Soluble Fas (sFAS) can antagonise cell surface FasL by competitive inhibition and so allow continued T-cell proliferation, a phenomenon shown to be significant in lymphoproliferative disorders such as non-Hodgkin’s lymphoma^109^.

This is the first time, to our knowledge, that evidence of such an extensively conserved systemic immune response to these infectious agents, directly comparable to that seen in many human and animal models of mycobacterial disease, has been demonstrated in the cat. This suggests that the occurrence of mycobacterial infections in this species could act as a naturally occurring model of human disease. This capacity could be particularly significant in overcoming the known shortcomings of murine models of mycobacterial disease, particularly as feline cases occur with relatively high clinical frequency and so could provide a naturally-occurring reliable source of study data.

Within the variety of mycobacterial species which are known to infect cats; those belonging to the MTBC, and *M*. *bovis* in particular, pose the greatest risk of zoonotic spread to in-contact humans^39–41^. Currently the gold standard diagnostic test to identify these organisms which can threaten human health is mycobacterial culture; however, *M*. *bovis* takes a minimum of 6–8 weeks to confirm by culture and *M*. *microti* requires even longer at 12–16 weeks^9,14,42^. Within the cytokines analysed in this study; Flt-3L, GM-CSF and IL-2 were all significantly elevated in cats with a diagnosis of infection with an MTBC organism compared to all other groups. Therefore, it may be possible that these cytokines in particular could be used to more rapidly identify those infections that pose zoonotic risk to owners and the general public, allowing for earlier intervention.

Cytokine profiling in companion animal medicine has been the subject of a number of recent studies^62,64,65^. One limitation of such studies to date is the study design to compare cytokine profiles in animals with the designated diagnosis of interest and apparently healthy animals as controls. In our investigations, we additionally included a small number of cats that had been hospitalised for reasons unrelated to mycobacterial disease. This allowed us to identify that the changes in concentration of the cytokines KC and IL-8 were not specific to mycobacterial infection, but rather may simply occur when cats were ill or stressed. These findings indicate that including such a group in experimental design is and will be important for the assessment of diagnostic accuracy, including sensitivity and specificity, of cytokine profiling in the future. Greater accuracy would be achieved by expanding on our small sample size (n = 6) and also by matching the diagnoses in this group to those with similar aetiopathogenesis to the diagnosis of interest. For example, such a study could include cats with other infectious granulomatous disease such as feline infectious peritonitis (FIP), where multiplex expression profile analysis of serum samples have also shown elevations in RANTES and GM-CSF concentrations^64^.

A limitation of the study we report here is its retrospective nature; this meant that it was not possible to have a standardised diagnostic assessment of each cat and samples from each cat did not undergo the gold standard test of mycobacterial culture. The majority of cats in this study were diagnosed by IGRA which has been shown to have a higher sensitivity for MTBC than NTM infections, which may have introduced some bias towards MTBC infected individuals in our population^45^. Similarly, the amount of time elapsed between the time of infection, the infective dose received, and the time of blood sampling was not known for these cats. It is therefore possible that the changes seen could reflect different stages or magnitude of the feline response to mycobacteria; for example, MTBC infections may be diagnosed earlier in the course of infection than NTM infections and so the differences seen may simply reflect an early immune response. It was therefore not possible for us to generate sensitivity or specificity data for these cytokines nor to assess their diagnostic capacity in combination as has been achieved in human medicine. To further investigate the potential for cytokine profiling in feline mycobacterial disease, prospective recruitment of cases would allow for additional control over the time of blood sampling in relation to patients being presented to a veterinary surgeon. Such a study would additionally further allow changes in cytokine concentrations to be tracked over time, and in response to treatment. This would allow much greater accuracy regarding the cessation of antimicrobial therapy in these cases, which is not currently possible.

## Conclusion

In this study, we demonstrate a conserved immunological response to mycobacteria by domestic cats analogous to that of other species. Further research into feline tuberculosis as a spontaneously occurring model of a significant human disease is urgently required. These data show that cytokine profiling has the practical and immunological potential to be developed as a sensitive and specific diagnostic test for the presence of mycobacteria in feline infections, to readily speciate them and help inform on zoonotic risk to exposed humans. The Milliplex MAP Feline Cytokine Magnetic Bead multiplex assay was shown to be a practicable and reliable platform for these measurements and further work should focus on the prospective diagnostic utility of the findings reported in this study.

## Electronic supplementary material

Supplementary Information
