# Supplementary material for: Cytokine and Chemokine Concentrations as Biomarkers of Feline Mycobacteriosis
Source: Sci Rep. 2018 Nov 23;8:17314. doi: 10.1038/s41598-018-35571-5 (PMC6251861; doi:10.1038/s41598-018-35571-5)
Supplement: Supplementary file 1 — Supplementary Information [file 41598_2018_35571_MOESM1_ESM.docx]

Cytokine and Chemokine Concentrations as Biomarkers of Feline Mycobacteriosis

O’Halloran, C.^1*^, McCulloch, L.,^2^ Rentoul, L.^3^, Alexander, J.^3,^ Hope, J.C.,^1a^ and Gunn-Moore, D.A.^1a^

^1^Royal (Dick) School of Veterinary Studies and The Roslin Institute, University of Edinburgh, Easter Bush Campus, Edinburgh, Scotland, EH25 9RG.

^2^UK Dementia Research Institute, Edinburgh Medical School, University of Edinburgh, University of Edinburgh, Scotland, EH16 4SB.

^3^ MilliporeSigma (a Division of Merck KGaA, Darmstadt, Germany), 3050 Spruce Street, St. Louis, MO, USA.

^4^Waltham Centre for Pet Nutrition, Leicestershire, UK.

^a^Joint last authors.

*Conor O’Halloran, BVSc MSc MRCVS: conor.o’halloran@roslin.ed.ac.uk

**Supplementary Data**

**Table I: Cytokine concentrations detected for each group of cats; healthy controls, cats infected with *Mycobacteria spp.* and cats hospitalised for other reasons.** All concentrations are given in pg/ml. Rows in bold denote a statistically significant difference between at least two of the three groups (Kruskal-Wallis test by ranks p<0.05)**.** Legend: N; total number of cats per group, SD; standard deviation.

| **Cytokine/**  **chemokine** | **Control group (N=16)**  Mean ± SD  (Reference interval) | **Mycobacteria infected (N=116)**  Median; range  (95% confidence interval) | **Hospitalised cats (N=6)**  Median; range  (95% confidence interval) | **Statistical power calculated *post-hoc*** |
| --- | --- | --- | --- | --- |
| **sFAS** | **33.09 ± 18.16**  **(24.18-41.89)** | **12.25; 0-191.92**  **(5.32-19.06)** | **32.17; 23.06-45.35**  **(28.73-47.07)** | **99%** |
| Flt-3L | 43.34 ± 15.37  (35.81-50.87) | 69.20; 20.76-223.63  (41.46-77.59) | 49.74  (2.44-473.41) | <50% |
| **GM-CSF** | **11.53 ± 4.65**  **(9.25-13.81)** | **24.82; 0-60.17**  **(19.19-29.37)** | **10.20; 0-13.72**  **(4.81-17.85)** | **91%** |
| IFN-γ | 71.59 ± 55.17  (44.55-98.62) | 76.93; 2.22-5499.63  (0-220.14) | 125.45; 34.29-300.30  (12.06-612.74) | <50% |
| **IL-1β** | **28.79 ± 9.09**  **(24.33-33.25)** | **18.97; 0-272.72**  **(15.74-32.19)** | **5.00; 0-7.83**  **(1.23-15.67)** | **88%** |
| **IL-2** | **0 ± 0**  **(0-0)** | **86.62; 0-1129.91**  **(22.65-135.28)** | **0; 0-0**  **(0-0)** | **>99.9%** |
| **PDGF-BB** | **0 ± 0**  **(0-0)** | **450.33; 0-1323.25**  **(371.34-529.31)** | **214.55; 0-267.54**  **(128.14-344.52)** | **>99.9%** |
| IL-12 (p40) | 157.16 ± 93.26  (111.47-202.86) | 359.62; 77.03-236.74  (100.91-460.51) | 358.32; 64.19-643.79  (53.01-1494.22) | <50% |
| **IL-13** | **44.40 ± 18.28**  **(35.44-53.36)** | **20.05; 2.29-118.58**  **(14.99-25.89)** | **7.17; 0-8.65**  **(1.74-12.97)** | **76%** |
| **IL-4** | **212.03 ± 183.34**  **(122.19-301.87)** | **9.38; 2.65-68.58**  **(3.82-14.90)** | **568.28; 74.05-2500.93**  **(38.95-6357.42)** |  |
| IL-6 | 65.84 ± 47.44  (42.59-89.10) | 429.86; 1.51-1938.58  (0-1184.39) | 37.31; 3.94-79.67  (3.57-208.10) | <50% |
| **IL-8** | **16.10 ± 17.61**  **(7.48-24.73)** | **137.80; 14.72-3644.62**  **(46.21-346.12)** | **50.95; 2.18-267.69**  **(4.55-232.43)** |  |
| **KC** | **3.18 ± 1.58**  **(2.41-3.95)** | **16.10; 1.21-624.47**  **(11.40-32.31)** | **125.99; 2.12-186.32**  **(0.00-2588.71)** |  |
| SDF-1 | 658.01 ± 265.07  (528.13-787.89) | 332.31; 39.34-2671.33  (50.68-705.69) | 304.70; 0-428.28  (122.73-671.10) | <50% |
| **RANTES** | **9.45 ± 3.72**  **(7.62-11.28)** | **19.28; 2.41-505.13**  **(16.21-51.97)** | **5.68; 0.23-5.36**  **(2.21-13.44)** |  |
| SCF | 74.03 ± 19.89  (64.28-83.77) | 75.14; 9.58-827.30  (53.14-106.28) | 66.27; 5.69-205.36  (32.21-151.14) | <50% |
| MCP-1 | 1497.87 ± 779.95  (1115.69-1880.04) | 1297.77; 37.62-13449.58  (341.74-1320.36) | 1348.69; 560.01-2142.26  (445.38-3369.35) | <50% |
| **TNF-α** | **34.41 ± 15.07**  **(27.02-41.79)** | **54.84; 10.57-1527.12**  **(51.25-128.27)** | **16.42; 0-18.82**  **(12.20-21.78)** |  |
| IL-18 | 200.80 ± 194.17  (105.66-295.94) | 133.40; 0-1932.34  (0-108.39) | 756.99; 539.00-974.28  (411.54-1314.89) | <50% |

**Table II: Cytokine concentrations detected for each sub-group of cats infected with *Mycobacteria spp.* designated into those cases infected with *M. bovis, M. microti* or non-tuberculous (NTM) mycobacteria.** All concentrations are given in pg/ml. Rows in bold denote a statistically significant difference between the groups (Mann Whitney U test, p≤0.003) between at least one of these groups and the healthy control cats (Table I). Legend: N; total number of cats per group.

| **Cytokine/**  **chemokine** | ***M. bovis-*infected cats N=22**  Median  (95% confidence interval) | ***M. microti­*-infected cats N=43**  Median  (95% confidence interval) | **NTM infected cats N=15**  Median  (95% confidence interval) | **Statistical power calculated *post-hoc*** |
| --- | --- | --- | --- | --- |
| sFAS | 8.26  (4.30-13.79) | 7.28  (2.28-12.58) | 15.70  (12.02-23.79) | <50% |
| **Flt-3L** | **80.79**  **(62.69-90.61)** | **73.54**  **(64.47-101.37)** | **42.87**  **(31.80-54.62)** | **94%** |
| **GM-CSF** | **27.63**  **(20.92-31.74)** | **27.88**  **(20.85-31.78)** | **11.25**  **(10.87-11.57)** | **83%** |
| IFN-γ | 89.74  (35.18-111.13) | 270.21  (79.63-337.83) | 52.58  (28.95-89.39) | <50% |
| IL-1β | 24.52  (21.69-26.28) | 27.93  (25.60-31.48) | 27.87  (27.14-76.00) | <50% |
| **IL-2** | **132.59**  **(115.52-155.35)** | **176.97**  **(149.59-185.48)** | **14.08**  **(2.04-17.07)** | **>99.9%** |
| **PDGF-BB** | **0**  **(0-0)** | **363.47**  **(148.47-511.47)** | **0**  **(0-0)** | **>99.9%** |
| **IL-12 (p40)** | **309.53**  **(289.67-646.77)** | **457.38**  **(332.72-550.28)** | **283.89**  **(200.40-486.63)** | **72%** |
| IL-13 | 40.74  (21.08-70.01) | 28.42  (23.87-59.31) | 14.16  (2.24-112.20) | <50% |
| IL-4 | 55.89  (23.66-146.82) | 154.55  (86.89-355.49) | 76.49  (20.08-137.54) | <50% |
| **IL-6** | **15.91**  **(5.65-28.98)** | **33.98**  **(20.51-99.72)** | **29.64**  **(9.08-64.30)** | **83%** |
| **IL-8** | **105.84**  **(59.86-215.20)** | **164.68**  **(51.89-201.06)** | **500.67**  **(137.47-818.24)** | **90%** |
| KC | 9.18  (2.39-20.12) | 15.16  (2.33-19.75) | 35.79  (1.88-97.68) | <50% |
| **SDF-1** | **2.88**  **(2.00-26.89)** | **20.41**  **(2.81-26.54)** | **20.28**  **(4.59-94.51)** | **89%** |
| **RANTES** | **43.18**  **(23.92-58.29)** | **27.18**  **(24.94-59.96)** | **45.24**  **(9.94-71.63)** | **87%** |
| SCF | 73.87  (56.86-103.04) | 192.57  (90.03-208.47) | 69.25  (43.43-90.25) | <50% |
| **MCP-1** | **472.40**  **(202.24-1946.22)** | **1136.32**  **(441.82-2398.05)** | **70.77**  **(54.21-193.52)** | **95%** |
| **TNF-α** | **162.47**  **(155.53-194.18)** | **41.58**  **(18.02-55.98)** | **12.84**  **(11.07-39.67)** | **98%** |
| IL-18 | 117.04  (114.00-222.90) | 45.62  (39.06-166.44) | 38.05  (22.97-147.84) | <50% |
